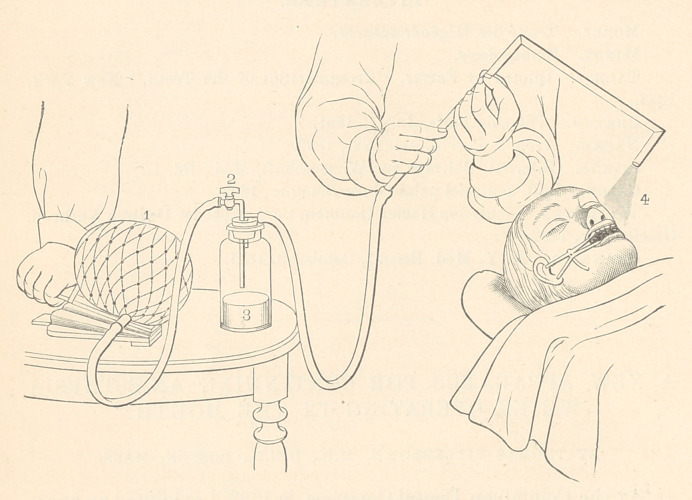# A New Apparatus for Continuing Anæsthesia While Operating in the Mouth

**Published:** 1895-12

**Authors:** Thomas Fillebrown

**Affiliations:** Boston, Mass.


### A NEW APPARATUS FOR CONTINUING ANÆSTHESIA WHILE OPERATING IN THE MOUTH.[1]

BY THOMAS FILLEBROWN, M.D., D.M.D., BOSTON, MASS.

At the Columbian Dental Congress, in 1893, I exhibited an apparatus for maintaining anæsthesia without a face-piece, which I described as consisting of "a bellows, connected by rubber tubing with the long tube of a twelve-ounce wash-bottle, with a stop-cock intervening to regulate the flow of air. From the bottle extends a half-inch rubber tube to the patient. The bottle is filled one-third full of ether. The bellows is inflated and the stop-cock opened, so as to allow the air to bubble up freely through the ether and become saturated with ether vapor. The etherized air is discharged through the second tube, a few inches from the patient's face."

I have since found it unnecessary to pass the air through the

[1] Read before the Academy of Stomatology, October 15, 1895.

anæsthetic, but obtain equally good results by simply passing the air over the ether or chloroform. I have also found that if the administration of the anæsthetic is somewhat prolonged, the evaporation reduces the temperature so low as to prevent saturation of the air; when this obtains, the hand of an assistant or a cloth wet with warm water should be applied to the bottle to raise the temperature to, at least, 60° F.

In cases of children I have found no difficulty in, from the first, maintaining a perfect anæsthesia, but my experience with adults proves that they must be first thoroughly anæsthetized by ordinary methods, as many adult patients require an almost entire exclusion of air for a time to become fully anæsthetized. I encountered two partial failures before I realized the cause. But with these patients, this method proved more than sufficient, after the anæsthesia was made complete by the ordinary administration. I have lately added a bent metallic delivery-tube, which enables the assistant to stand behind the patient while administering the anæsthetic, and thus be entirely out of the way of the operator. The mouth-gag I believe to be essential to success, and I never omit its use. Perhaps infants might not need it.

I have thus far been intent on establishing the complete success of the anæsthesia, and have made no effort to economize the ether.

As I now use it, four ounces of ether will maintain complete anæsthesia for one hour; further experience may make less sufficient.

With this apparatus, simple as it is, complete anæsthesia may be maintained for any length of time, and any operation on the face or within the mouth of the patient be performed, and the operator will not be interfered with any more than during an operation on any other part of the body. An assistant can use the sponge freely and keep the throat clear of blood and mucus, so that very seldom will it be necessary to use any other means to free the mouth of accumulations. The accompanying cut shows all the parts and will make the preceding explanation clear.

The bellows is one of the ordinary dentist's foot-bellows. The bottle, with rubber stopper, is one taken from an oxygen apparatus found at the dental depots. The stop-cock is not absolutely essential, and some glass tubing will answer to pass the air through the wash-bottle. A tin-worker can furnish the delivery-tube at short notice, and rubber tubing is always at hand, so no one need be deprived of the benefits of this method for lack of an apparatus.

The following twenty-three cases show, I think, sufficient experience with the apparatus to establish the fact of its efficiency and usefulness. Eighteen of these were cases in my own private and hospital practice.

Case I.—May, 1893. A student in the Harvard Dental School, who kindly consented to be an experimental subject. He was anæsthetized to complete insensibility in ten minutes, without a mouth-gag. He was both willing and able to keep his mouth open.

The mouth-gag was used in all the following cases, after the patient was etherized by the ordinary method.

Case II.—Male, aged twenty-five years. Removal of non-erupted third molar. Complete anæsthesia maintained twenty minutes.

Case III.—Male, aged twenty-six years. Operation on lip. Anæsthesia maintained twenty-five minutes.

Case IV.—Male, aged twenty-two years. Removal of necrosis of inferior maxilla. Anæsthesia maintained twenty minutes.

Case V.—Female, aged six years. Operation, uranoplasty. Anæsthesia maintained one hour and three-quarters.

Case VI.—Male, aged eleven years. Operation, uranoplasty. Anæsthesia maintained forty-five minutes.

Case VII.—Male, aged ten years. Operation, uranoplasty. Anæsthesia maintained one and one-half hours.

Case VIII.—Male, aged eleven years. Operation, staphylorrhaphy. Anæsthesia maintained one and three-quarters hours.

Case IX.—Male, aged twenty-nine years. Operation, reshaping flat nostril. Anæsthesia maintained thirty minutes.

Case X.—Male, aged twenty-four years. Operation, staphylorrhaphy. Anæsthesia maintained one hour.

Case XI.—Female, aged seven years. Operation, staphylorrhaphy. Anæsthesia maintained one hour and twenty minutes.

Case XII.—Female, aged six years. Operation, closure of hard and soft palates. Anæsthesia maintained one hour and ten minutes.

Case XIII.—Female, aged sixty years. Operation, removal of encysted cuspid. Anæsthesia maintained eighteen minutes.

Case XIV.—Female, aged six years. Operation, uranoplasty. Anæsthesia maintained thirty-two minutes.

Case XV.—Female, aged six and one-half years. Operation, staphylorrhaphy. Anæsthesia maintained fifty-five minutes.

Case XVI.—Female, aged forty years. Operation, exsection of right inferior maxillary nerve. Clinic before the New England Dental Society, at the Boston Dental College Hospital. Anæsthesia maintained thirty-two minutes.

Case XVII.—Male, aged forty years. Operation, staphylorrhaphy, at the Maine General Hospital, by Professor Weeks. Anæsthesia maintained one and one-quarter hours.

This patient was not fully anæsthetized when the operation was begun, and at first the apparatus was insufficient to maintain the insensibility; but after the patient was fully etherized it was more than equal to the work, and several times the tube had to be removed on account of too deep narcosis.

In one other case I attempted its use when the patient was only partially narcotized, and I had the same trouble; consequently I am led to doubt if it is practical to use the apparatus until the narcosis is complete.

Case XVIII.—Male, aged ten years. Operation, removal of tonsils by means of the cold wire snare. A clinic before the New England Dental Society, at the Oral Hospital of the Boston Dental College, by Professor George F. Eames. Anæsthesia maintained fifteen minutes.

Case XIX.—At same clinic, a male aged ten years. Operation, removal of adenoid growths, by Professor Eames. Anæsthesia maintained ten minutes.

The following cases, Nos. XX., XXI., XXII., XXIII., were operated on at the Lynn Hospital, Dr. C. M. Smith directing the maintaining of the anæsthesia.

Case XX.—Male, aged forty-five years. Sarcoma of antrum.

Operated on by Drs. Stevens and Smith. Anæsthesia maintained fifty minutes. "Anæsthesia complete during the whole time."

CASE XXI.—Necrosis of the ramus, including the angle of the under jaw. Operation by Drs. Pinkham and Smith. Anæsthesia maintained one hour.

CASE XXII.—Female, aged eleven years. Removal of adenoids. Operation by Dr. Stevens. Anæsthesia maintained fifteen minutes.

CASE XXIII.—Male, aged forty-five years. Sarcoma of antrum. Secondary operation by Dr. Stevens on Case XX. Anæsthesia maintained twenty minutes.

Many conceive the idea that it is a spray which is furnished the patient to breathe, but nothing is further from the truth. If a patient inhales from a sponge or towel saturated with ether, he does not breathe ether as such, but ether vapor. It is just the same if air is forced through or over liquid ether. The air simply takes up the ether vapor, and the patients breathe etherized air, the same as is breathed through a sponge or other inhaler.

The essential merit of my invention is that the etherized air is discharged towards the patient from a point far enough from the face to prevent the apparatus from interfering with the operation going on in the mouth, and in sufficient quantity and with sufficient force to furnish an anæsthetic atmosphere for the patient to breathe without taking in air from outside the current. I will add here that the surplus anæsthetic discharged into the atmosphere will not sensibly affect either the operator or his assistant.

Air containing ether or chloroform vapor has long been used for inducing anæsthesia, but always with an inhaler that covered the face and rendered any operation about the mouth impossible while the anæsthetic was being inhaled. Dr. Snow, in 1849, mixed chloroform vapor with air in the definite proportion of three and one-half per cent., and found it very safe, successful, and economical. Clover, in 1862, used the same mixture as Snow, and devised an apparatus for administering it, the principal feature of which was a large reservoir-bag hung over the operator's shoulder. Snow, about the same time, devised an inhaler lined with lint. The lint was wet with chloroform, and through this the air was drawn by inhalation, which, while passing over the surface of the lint, took up a portion of the vapor.

Dr. Horace Packard, of Boston, a few years since, devised a very convenient and compact apparatus for administering etherized air, which was suggested to him by the Junker system for giving chloroform, and it was the use of this that suggested to me the apparatus which I have described. My object in writing this article is to record the success which has attended its use in my own practice and that of others, and to further and more completely illustrate its mechanism, that its simplicity and usefulness may be better understood.

## Figures and Tables

**Figure f1:**